# Mycoidesin, a novel lantibiotic, exhibits potent bacteriostatic activity against *Listeria monocytogenes* and effectively controls its growth in beef

**DOI:** 10.1128/aem.00067-25

**Published:** 2025-03-25

**Authors:** Fei Zhang, Jiajia Ding, Shu Liu, Guoqiang Huang, Shulin Deng, Mengyu Gao, Hualin Liu, Wanjing Lv, Xin Zeng, Bingyue Xin, Congcong Xu

**Affiliations:** 1 Anhui Province Key Laboratory of Pollutant Sensitive Materials and Environmental Remediation, College of Life Sciences, Huaibei Normal University 58286 , Huaibei, Anhui, China; 2 National Key Laboratory of Agricultural Microbiology, Huazhong Agricultural University 47895 , Wuhan, Hubei, China; 3 Guangdong Perfect Life Health Science and Technology Research Institute Co. Ltd, Zhongshan, Guangdong, China; 4 School of Life Sciences, Qufu Normal University 56650 , Qufu, Shandong, China; Anses, Maisons-Alfort Laboratory for Food Safety, Maisons-Alfort, France

**Keywords:** *Listeria monocytogenes*, biopreservatives, bacteriocin, *Bacillus mycoides*, mycoidesin

## Abstract

*Listeria monocytogenes* can cause severe listeriosis, with the consumption of contaminated food being an important route of its transmission. Biopreservatives can be used for the prevention and control of *L. monocytogenes* in food. In this study, we identified a novel lantibiotic, mycoidesin, with potent bacteriostatic activity against *L. monocytogenes*. It exhibited 4- to 16-fold higher bacteriostatic activity against the *L. monocytogenes* strains than nisin A. Analysis of the mode of action of mycoidesin revealed that it exerted bacteriostatic activity against *L. monocytogenes* ATCC 19111 at low and high concentrations (1×–32× MIC, 0.39–12.5 µM). It blocked cell wall synthesis by binding to Lipid II and inhibiting the growth of *L. monocytogenes*. For other sensitive strains, such as *Bacillus cereus* CMCC 63301, mycoidesin exerted a bacteriostatic effect at a low concentration (1× MIC, 1.56 µM) via the same mechanism, whereas it exerted a bactericidal effect at high concentrations (2×–8× MIC, 3.13–12.5 µM), which can damage the cell membrane and cause cell death. The stability test showed that mycoidesin had increased stability compared to nisin A. Additionally, mycoidesin showed low cytotoxic and hemolytic activity. Furthermore, mycoidesin effectively inhibited the growth of *L. monocytogenes* in beef and delayed the decline in beef quality. Our study demonstrates the potential of mycoidesin as a biopreservative to prevent *L. monocytogenes* contamination and improve the safety of meat and meat products in the food industry.

**IMPORTANCE:**

This study aimed to identify highly effective, stable, and safe natural bacteriocin preservatives with anti-*Listeria monocytogenes* activity. We isolated a novel class II lantibiotic, mycoidesin, which exhibited more efficient bacteriostatic activity against *L. monocytogenes* and increased stability compared to the applied bacteriocin food preservative, nisin A. Mycoidesin also showed favorable biosafety. Moreover, mycoidesin could be effectively used for controlling *L. monocytogenes* in beef, demonstrating its potential as a biopreservative to prevent *L. monocytogenes*-related contamination and improve the safety of meat and meat products in the agricultural and food industries.

## INTRODUCTION

*Listeria monocytogenes* is an important food-borne pathogen that causes gastroenteritis in immunocompetent individuals, systemic listeriosis in immunocompromised patients, abortion in pregnant women, and neonatal listeriosis in newborns (1, 2). Although the incidence of *L. monocytogenes* infection is relatively low, high hospitalization and mortality rates have been recorded; 2,738 cases of listeriosis occurred in the European Union countries, with 1,330 hospitalizations and 286 deaths in 2022 (3–6). Approximately 1,600 listeriosis cases with a 20%–30% mortality rate have been reported in the United States (7). In China, 253 cases of listeriosis, with 25.7% mortality, were reported between 2011 and 2016 (8). The strict control of *L. monocytogenes* in food has led to the recall of contaminated food, causing significant economic losses of approximately 2.8 billion dollars annually (1).

Consuming food contaminated with *L. monocytogenes* is a critical cause of sporadic infections and listeriosis outbreaks (9). Food preservatives can inhibit the growth of microorganisms, prevent spoilage, and extend the shelf life of food. Chemical preservatives are cost-effective and beneficial; however, their misuse may cause damage to human health, such as allergic reactions and carcinogenicity (10, 11). Natural food preservatives, such as bacteriocins, which are antimicrobial peptides produced by certain bacteria and synthesized by ribosomes, exhibit potent antimicrobial activities against food-borne pathogens, do not affect the sensory qualities of the food, are non-toxic to humans, and are widely accepted by consumers (12, 13). Lantibiotics are a type of best-characterized bacteriocins that possess unusual amino acids, such as lanthionine, 3-methyllanthionine, dehydroalanine, and dehydrobutyrine (14). Nisin, the first lantibiotic discovered, exhibits potent activities against many food-borne pathogens, such as *Clostridium botulinum*, *Staphylococcus aureus*, and *L. monocytogenes*; it has been widely used as a bacteriocin preservative in over 50 countries (12). However, its low stability and solubility in neutral and alkaline conditions limit its application (15, 16). Therefore, there is an urgent need to develop new bacteriocins with better properties.

The *Bacillus cereus* group consists of more than two dozen closely related species, such as *Bacillus cereus*, *Bacillus mycoides*, *Bacillus thuringiensis*, and *Bacillus wiedmannii* (17). Recently, over 20 different types of bacteriocins have been identified in this group, some of which exhibit antimicrobial effects against many food-borne pathogens (18). However, the *B. cereus* group contains several types of novel bacteriocins, many of which have not yet been identified or characterized in genome mining studies (19, 20).

In this study, we used *L. monocytogenes* as an indicator strain to screen strains of the *B. cereus* group from soil samples collected from various regions and habitats across China. We identified a novel lantibiotic, mycoidesin, produced by *B. mycoides* LX30, with potent anti-*L*. *monocytogenes* activity. The structure, antimicrobial activity, antimicrobial mechanism, stability, biosafety, and application of mycoidesin in beef preservation were determined.

## MATERIALS AND METHODS

### Screening for bacteriocin-producing strains

Using *L. monocytogenes* ATCC19111 as an indicator strain, we screened 132 strains of the *B. cereus* group, isolated from the soil of various regions and habitats across China. These strains were identified as the *B. cereus* group via 16S rRNA sequence analysis and preserved in our laboratory for the detection of antimicrobial activities. Each strain was activated for 12 h in the TSB medium, and the culture (1 mL) was transferred into the TSB medium (100 mL) and incubated for 24 h at 25°C with shaking at 200 rpm. Culture aliquots (1 mL) were collected, the absorbance was measured at 600 nm, and the antimicrobial activity of the fermentation supernatant was detected using the agar diffusion method (21). Briefly, 20 mL TSB agar (approximately 43°C) containing 5 × 10^5^ colony-forming units (CFUs)/mL *L. monocytogenes* ATCC19111 cells was spread onto a plate. After the medium solidified, wells were punched, and 50 µL of cell-free supernatant was added. The plate was placed at 4°C for 2 h and then at 30°C for 16 h to observe the inhibition zone. The supernatant with antimicrobial activity was incubated with mixed protease (1 mg/mL of α-chymotrypsin and trypsin) at 30°C for 3 h, and the residual antimicrobial activity of the supernatant was detected using the agar diffusion method. Cell-free supernatants of *B. mycoides* LX30 showed the highest antimicrobial activity against *L. monocytogenes* ATCC19111, and the antimicrobials were degraded by proteases. Thus, *B. mycoides* LX30 was considered a bacteriocin-producing strain and was further studied.

### Genome sequencing and bacteriocin gene cluster analysis

*Bacillus mycoides* LX30 was sent to Novogene Bioinformatics Technology Co., Ltd. for whole-genome sequencing using the HiSeq PE150 and Oxford Nanopore platforms. The reads of *B. mycoides* LX30 were assembled into a complete genome using the PGCGAP version 1.0.35 software (22). AntiSMASH and BAGEL4 were used to predict and analyze the bacteriocin gene cluster of *B. mycoides* LX30 (23, 24). The BLASTp software was used to analyze the function of each putative protein in the mycoidesin gene cluster.

### Purification of the antimicrobial substance of *B. mycoides* LX30

A single colony of *B. mycoides* LX30 was picked and grown in 100 mL of TSB medium at 30°C, 200 rpm for 12 h. The culture was transferred to 10 L of TSB medium and cultured at 30°C, 200 rpm for 8 h. The fermentation broth was centrifuged (12,000 × *g* for 10 min at 4°C), and the acquired supernatant (10 L) was mixed with 1 L Amberlite XAD-7HP (Sigma-Aldrich, USA) at 4°C for 6 h with shaking. The resin was washed with 5 L ddH_2_O and 2.5 L 30% (vol/vol) ethanol sequentially, and the adsorbed antimicrobial agents were eluted with 1 L 80% (vol/vol) ethanol (pH 2.0). The eluent was concentrated to 10 mL at 45°C via a rotary evaporator and then centrifuged (10,000 × *g* for 5 min). The supernatant was collected as the antimicrobial crude extract (CE).

The *B. mycoides* LX30 CE was further analyzed using high-performance liquid chromatography (HPLC; WUFENG LC-100 system, China). The mobile phases used were acetonitrile and ddH_2_O (0.1% trifluoroacetic acid). The CE (100 µL) was loaded and separated on a C18 column (Agilent, USA) using a linear gradient of 10%–90% acetonitrile for 30 min at a flow rate of 1 mL/min. The effluent was monitored at 220 nm and collected every minute, and its antimicrobial activity was detected using the agar diffusion method. Fractions exhibiting antimicrobial activity were repeatedly collected and then lyophilized using a vacuum freeze dryer. The obtained powder was weighed (23.5 mg) and stored at −20°C for further study.

### LC-MS and LC-MS/MS analyses of the antimicrobial substance of *B. mycoides* LX30

Liquid chromatography-mass spectrometry (LC-MS) analysis of mycoidesin was performed using an Agilent 6540 Ultra-High Definition Accurate-Mass Quadrupole Time-of-Flight LC-MS system. The conditions for MS analysis included positive ion mode, 9 L/min of drying gas flow, 35 psig of nebulizer pressure, 350°C of capillary temperature, and 3.5 kV of source voltage. The structure of anti-*L*. *monocytogenes* substance was analyzed using LC-MS/MS, and the fragment was obtained by collision-induced dissociation (25 V of collision energy).

### Determination of the MICs and MBCs of mycoidesin

The microdilution method (25) was used to measure the MIC of mycoidesin against indicator strains (Table S2). HPLC-purified mycoidesin was weighed and dissolved in a liquid culture medium to prepare various concentrations of mycoidesin (200.00, 100.00, 50.00, 25.00, 12.50, 6.25, 3.13, 1.56, 0.78, 0.39, 0.20, and 0.10 µM). A volume of 50 µL of different concentrations of mycoidesin solutions and 50 µL culture (1 × 10^6^ CFU/mL) of the indicator strain were mixed in 96-well plates. The final concentrations of mycoidesin were 100.00, 50.00, 25.00, 12.50, 6.25, 3.13, 1.56, 0.78, 0.39, 0.20, 0.10, and 0.05 µM. The plates were incubated at 30°C for 16 h, and the lowest concentration at which no growth was observed was defined as the minimum inhibitory concentration (MIC). The culture that showed no visible bacterial growth was inoculated on agar plates and incubated for 24 h. The minimum bactericidal concentration (MBC) was the lowest concentration that killed 99.9% of the bacterial population. Nisin A was purified (Cayman Chemical, USA) as previously described (26) and was used to measure and compare the MIC values of *L. monocytogenes* strains and mycoidesin.

### Mode of action of mycoidesin

#### Intracellular accumulation of UDP-MurNAc-pentapeptide

*Bacillus cereus* CMCC63301 and *L. monocytogenes* ATCC19111 were grown to an OD_600_ of 0.3 and incubated with chloramphenicol (130 µg/mL) for 15 min. Mycoidesin (1× and 2× MIC) and vancomycin (10× MIC; positive control) were added and incubated for 60 min. The cells were collected, added to boiling water, treated for 15 min, centrifuged, and the supernatant was lyophilized. The intracellular accumulation of the peptidoglycan precursor UDP-MurNAc-pentapeptide was analyzed using HPLC (Hypersil ODS column) and identified via mass spectrometry (27).

#### Time-kill curves

*Bacillus cereus* CMCC63301 and *L. monocytogenes* ATCC19111 were cultured in TSB medium to an OD_600_ of 0.3 and exposed to mycoidesin of 1× MIC (*Lm*, 0.39 µM; *Bc*, 1.56 µM), 2× MIC (*Lm*, 0.78 µM; *Bc*, 3.13 µM), 4× MIC (*Lm*, 1.56 µM; *Bc*, 6.25 µM), 8× MIC (*Lm*, 3.13 µM; *Bc*, 12.50 µM), 16× MIC (only applied to *Lm*, 6.25 µM), and 32× MIC (only applied to *Lm*, 12.5 µM) at 30°C for 3 h. Cell growth was monitored hourly by measuring CFU on TSB plates and OD_600_ values of the cultures.

#### Detection of potassium release

Aliquots of 10 mL *L*. *monocytogenes* ATCC19111 cell suspensions (OD_600_ = 0.3) were treated with 1× MIC (0.39 µM), 8× MIC (3.13 µM), and 32× MIC (12.5 µM) of mycoidesin, and *B. cereus* CMCC63301 cell suspensions were treated with 1× MIC (1.56 µM), 2× MIC (3.13 µM), and 4× MIC (6.25 µM) of mycoidesin for 1 h. The amount of potassium released was measured using a HI96750 Potassium Photometer.

#### SEM

*Bacillus cereus* CMCC63301 and *L. monocytogenes* ATCC19111 were grown in TSB medium to an OD_600_ of 0.3. *B. cereus* cells were treated with mycoidesin of 2× MIC (3.13 µM) and 4× MIC (6.25 µM) for 3 h at 30°C, and *L. monocytogenes* cells were treated with mycoidesin of 8× MIC (3.13 µM) and 32× MIC (12.5 µM) for 3 h at 30°C. The cells were harvested after centrifugation (6,000 × *g*, 10 min), washed with PBS buffer thrice, and fixed with 2.5% (vol/vol) glutaraldehyde for 12 h at 4°C. Subsequently, the cells were subjected to gradient dehydration for 10 min, treated with isoamyl acetate for 10 min, dried with a lyophilizer for 12 h, coated with gold-palladium, and observed using a Zeiss Gemini360 instrument (18).

### Cytotoxicity and hemolysis of mycoidesin

The cytotoxicity of mycoidesin was assessed in mouse embryonic fibroblast (NIH/3T3) cells using the Cell Counting Kit-8 (CCK-8) assay. NIH-3T3 cells were cultured in 96-well plates at 37°C in 5% CO_2_ for 24 h, and PBS with varying final concentrations of mycoidesin (1, 5, and 25 µM) was added and incubated at 37°C for 24 h. The CCK-8 solution was added and incubated for 3 h, and the OD_450_ was measured to calculate the percentage of viable cells. PBS and paclitaxel (Sigma-Aldrich, USA) were used as negative and positive controls, respectively.

The defibrillated sheep blood was centrifuged (5,000 × *g* for 5 min at 4°C), and the precipitated cells were washed and resuspended in PBS buffer. Then, 50 µL of PBS buffer with varied final concentrations of mycoidesin (1, 5, and 25 µM) was mixed with 50 µL of suspension of red blood cells into the 96-well plate and incubated at 37°C for 1 h. The sample was centrifuged (1,000 × *g* for 5 min at 4°C), and the absorbance of the supernatant was measured at 405 nm. PBS and Triton X-100 (1%) were used as negative and positive controls, respectively (28, 29).

### Stability of mycoidesin

Mycoidesin (6.25 µM, 4× MIC) and nisin A (12.5 µM, 4× MIC) solutions were adjusted to pH 7.0 and 8.0 and incubated at 37°C and 60°C for 12 h, respectively. After treatment for 2, 4, 6, 8, 10, and 12 h, the residual antimicrobial activity of the samples was measured using the agar diffusion method, with *B. subtilis* CMCC63501 as the indicator strain.

### Antimicrobial application of mycoidesin in beef

Tenderloin of beef (purchased from Darifa Supermarket, Huaibei) was cut into 3 × 3 cm pieces (10 ± 0.5 g). Each side of the beef was irradiated with ultraviolet radiation in a sterile ultra-clean platform for 30 min, inoculated with 100 µL of 10^5^ CFU/mL *L*. *monocytogenes* ATCC19111 on the beef surface, and then air dried for 30 min. Subsequently, the beef samples were immersed in the mycoidesin solution (0.39 µM, 1× MIC; 1.56 µM, 4× MIC; and 3.13 µM, 8× MIC) for 10 min and then air dried for 30 min. Beef samples were wrapped in sterile polyethylene bags, stored at 4°C, and subjected to various measurements on days 0, 3, 6, 9, 12, and 15. Sterile saline was used as the control.

To determine the total colony count of *L. monocytogenes*, beef samples (10 g) were mixed with 90 mL sterile saline and homogenized. The obtained suspension was spread on TSB agar, cultured at 30°C for 24 h, and the *L. monocytogenes* colonies were counted. The obtained suspension was centrifuged (10,000 × *g* for 10 min), and the pH values were measured using a pH meter. The steam distillation method was used to determine the total volatile basic nitrogen (TVB-N). The sample suspension was filtered through filter paper, and steam was distilled using a Kjeldahl apparatus (K-375; Buchi). The TVB-N values of samples were calculated based on hydrochloric acid (0.01 M) consumption. For sensory evaluation, a sensory evaluation team (comprising 10 people) was established based on the color, smell, and texture of beef. All assessors had a professional background in food for sensory evaluation and were not part of our team. Sensory training was provided to all the assessors before the experiment; they assessed each characteristic and scored individually. The highest sensory evaluation score of 10 represented the best sensory quality, 5–10 was considered acceptable, and the lowest score of 0 represented the lowest perceived quality, which was unacceptable.

### Statistical analysis

All experiments were conducted three times, and results were expressed as mean ± standard deviation. Student’s *t*-test was performed using SPSS 22.0 to determine statistical significance, which was set at *P* ≤ 0.05.

## RESULTS

### Screening the bacteriocin-producing strain *B. mycoides* LX30

**Fig 1:**
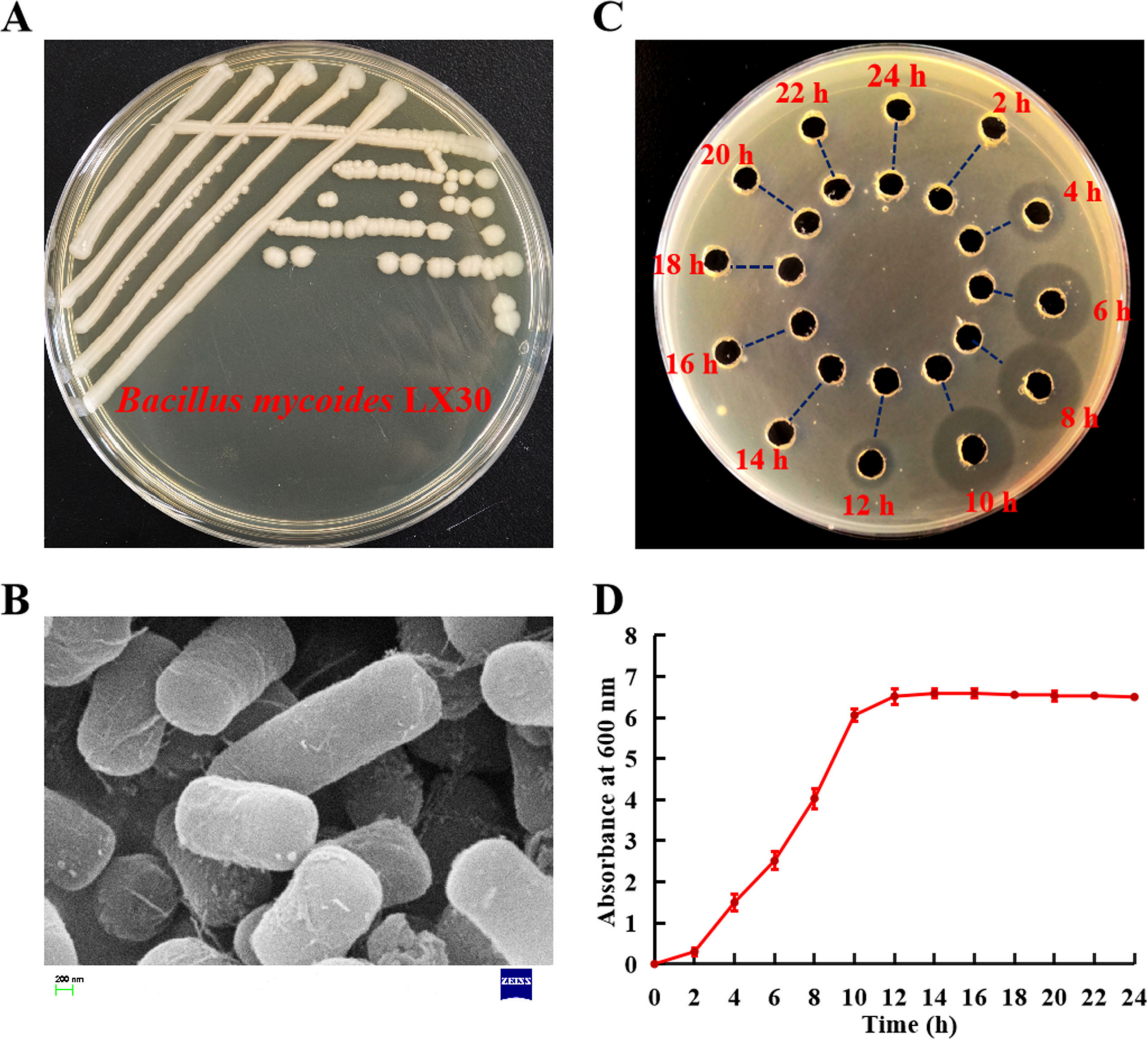
Screening of the bacteriocin-producing strain *Bacillus mycoides* LX30. (A) Colony morphology of *B. mycoides* LX30. (B) Scanning electron microscope observation of *B. mycoides* LX30. (C) Antimicrobial activity of the supernatant of *B. mycoides* LX30 against *Listeria monocytogenes* ATCC19111. Outer well: the fermentation supernatant; inner well: the fermentation supernatant treated with mixed enzyme. (D) Growth curve of *B. mycoides* LX30 in TSB medium.

Overall, 132 strains from the *B. cereus* group were screened for anti-*L*. *monocytogenes* activity. Of these, 13 strains showed antimicrobial activity against *L. monocytogenes* ATCC19111 (data not shown), with *B. mycoides* LX30 demonstrating the most potent antimicrobial activity. The antimicrobial substance of LX30 was produced in the logarithmic growth phase (4–12 h) (Fig. 1). The production of antibacterial substances began after 4 h of growth, the maximum inhibitory amount was recorded at 10 h, and the antimicrobial activity disappeared after 12 h of growth. The antimicrobial substance could be degraded by proteases, implying it was a bacteriocin. Therefore, the LX30 strain and its bacteriocins were subsequently analyzed.

### Genomic sequencing and analysis of the bacteriocin gene cluster of *B. mycoides* LX30

**Fig 2:**
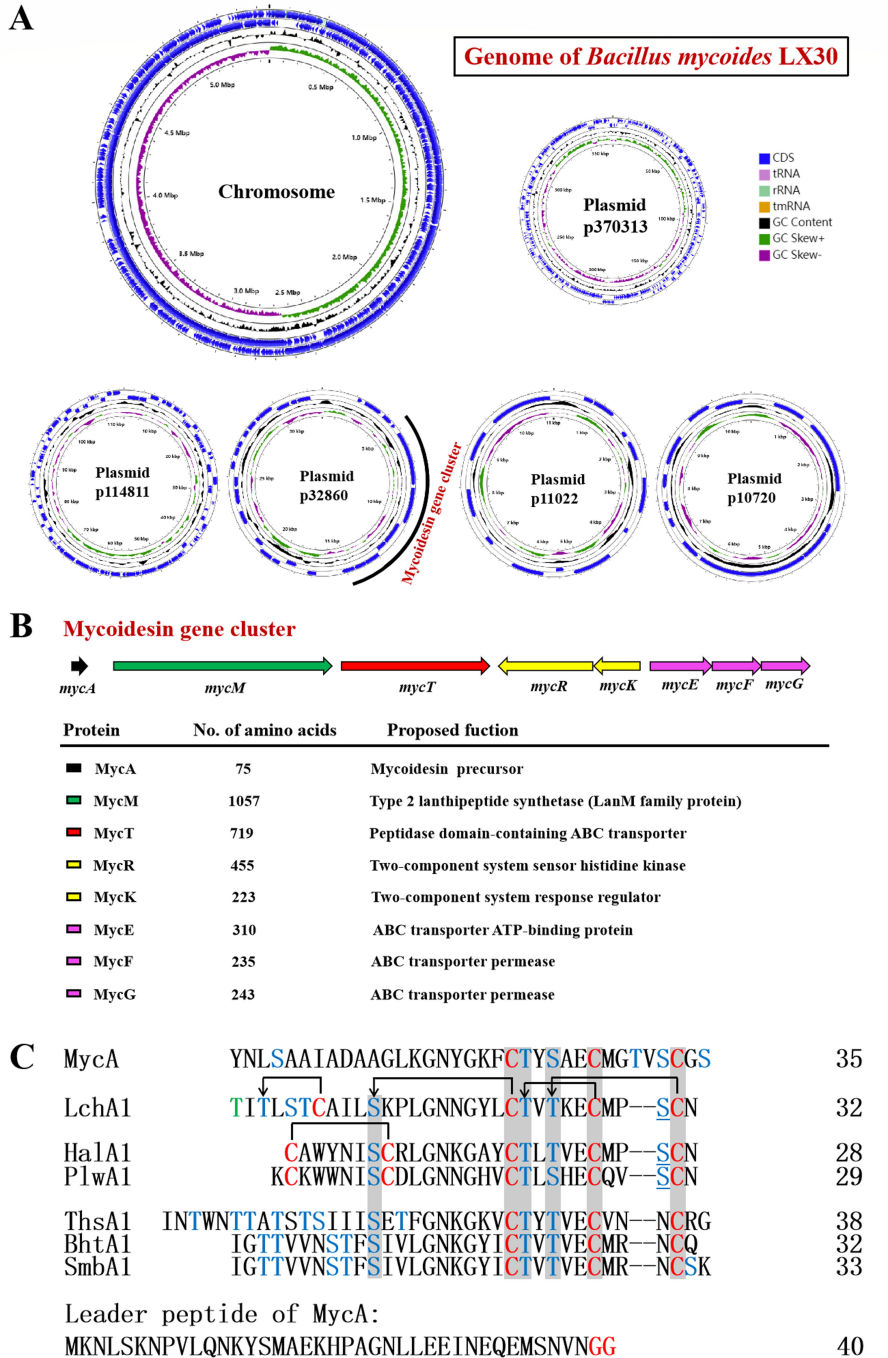
Gene cluster and precursor peptide of mycoidesin. (A) Genome of *Bacillus mycoides* LX30. (B) The gene cluster for mycoidesin biosynthesis in plasmid p32860 of *B. mycoides* LX30. (C) Multiple sequence alignment of MycA with precursor peptides of the lantibiotics lichenicidin VK21 (LchA1, ADM36018.1), haloduracin (HalA1, BAB04173), plantaricin W (PlwA1, AAG02567), thusin (ThsA1, ANP43731.1), BHT (BhtA1, AAZ76603), and Smb (SmbA1, BAD72777). Ser/Thr residues are indicated in cyan, and unmodified residues are underlined. Post-translational modification of the residue to Obu is indicated in green. The Cys residues are indicated in red. The thioether and disulfide bonds of LchAα and Halα peptides are shown in lines with and without arrows.

To reveal the biosynthetic gene cluster of bacteriocins produced by LX30, the complete genome of *B. mycoides* LX30 was sequenced, and the putative bacteriocin gene cluster was analyzed. The genome of *B. mycoides* LX30 consists of one chromosome and five plasmids (Fig. 2A). The circular chromosome contains 5,462,895 bp with 35.5% GC content (GenBank accession number CP092833.1). The five plasmids p370313, p114811, p32860, p11022, and p10720 consist of 370,292, 114,800, 32,860, 11,021, and 10,719 bp, respectively (GenBank accession numbers CP092834.1 to CP092838.1).

The plasmid p32860 contains a putative lanthipeptide, named mycoidesin biosynthetic gene cluster, which consists of eight genes, encoding a precursor peptide (*mycA*), a type 2 lanthipeptide synthetase (*mycM*), a peptidase domain-containing ABC transporter, which could be involved in transport (*mycT*), a two-component regulatory system, which consists of sensor histidine kinase (*mycK*), a response regulator (*mycR*), and an ABC transporter, which could be involved in immunity (*mycE*, *mycF*, and *mycG*) (Fig. 2A and B). The precursor peptide, MycA (75 aa), comprises an N-terminal leader peptide (40 aa) of the double glycine type and the C-terminus of the core peptide (35 aa). MycA was most homologous to LchA1, a precursor peptide of the two-component lantibiotic lichenicidin VK21 (38.10% identity) (Fig. 2C).

### Purification and identification of mycoidesin

**Fig 3:**
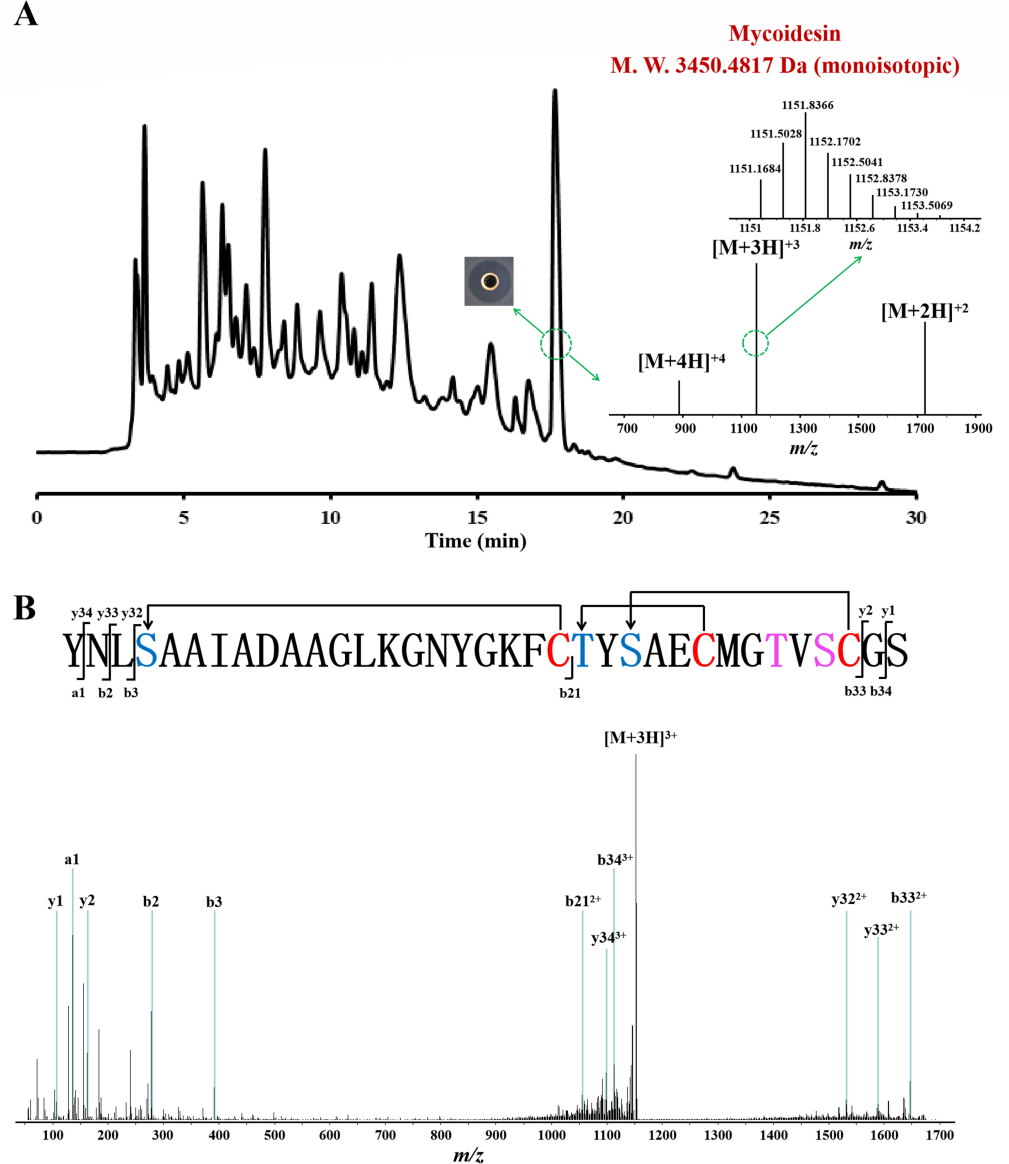
Purification and identification of the mycoidesin. (A) HPLC analysis of crude extracts of *B. mycoides* LX30 and MS analysis of mycoidesin. (B) MS/MS spectra and the proposed primary structure of mycoidesin. The fragment ions of mycoidesin are marked. The post-translationally modified Ser/Thr residues are presented in blue. One of the residues, Thr30, and Ser32 undergo a dehydration reaction; however, this is still uncertain based on the current data, and they are marked in purple. The Cys residues are indicated in red. The thioether bonds of mycoidesin are indicated by arrows.

*Bacillus mycoides* LX30 was cultured in TSB for 10 h, and the bacteriocin in the supernatant was adsorbed onto the resins and eluted to obtain the CE of the bacteriocin. CE was analyzed using HPLC, and only one fraction with a retention time of 17.66 min exhibited antimicrobial activity against *L. monocytogenes* ATCC19111; MS analysis revealed that this fraction contained a pure substance with a molecular mass of 3,450.4817 Da (monoisotopic signal) (Fig. 3A). The molecular weight of the predicted mature peptide of MycA, mycoidesin, was 3,522.5563 Da, which was higher than the measured mass of 72.0746 Da, implying that the dehydration reaction occurred in four amino acids, two serines, and four threonines (Fig. 3B). The detailed sequences of the antimicrobial substances were further analyzed using LC-MS/MS. The marked fragments of the antimicrobial substances coincided with the fragments of mycoidesin, thus proving that the antimicrobial substance LX30 is the lantibiotic mycoidesin (Fig. 3B; Table S1). There was no cleavage from Ser4 to Cys21 or from Thr22 to Cys33, confirming the presence of thioether crosslinks. The molecular fragment ions y1 measured 106.05 Da, indicating that Ser35 was not dehydrated. In addition, the molecular masses of fragment ions b21 and b33 revealed that either Thr30 or Ser32 was dehydrated. By combining the MS/MS data and the structures of LchA1, HalA1, PlwA1, ThsA1, BhtA1, and SmbA1, which exhibit amino acid sequence similarity to mycoidesin, the primary structure of mycoidesin was inferred (Fig. 3B).

### Antimicrobial spectrum and MICs and MBCs of mycoidesin

The antimicrobial activities of mycoidesin against various gram-positive and gram-negative bacterial strains were tested. The tested *L. monocytogenes* strains exhibited the highest susceptibility to mycoidesin (MIC values range from 0.20 to 0.78 µM) (Table S2), and compared with the applied food preservative Nisin A, mycoidesin demonstrated 4- to 16-fold higher bacteriostatic activity against the test *L. monocytogenes* strains (Table 1). The MBC values, including mycoidesin at high concentration (100 µM) for all test *L. monocytogenes* strains, were not determined (Table S2). Mycoidesin exhibited potent antimicrobial activity against other gram-positive strains, including *B. cereus*, *B. subtilis*, *Clostridium perfringens*, *Enterococcus faecalis*, and *Streptococcus suis*, with MIC values ranging from 1.56 to 6.25 µM and MBC values ranging from 1.56 to 12.5 µM. It showed modest antimicrobial activity against *Staphylococcus aureus* and *Staphylococcus epidermidis*, with MIC values of 25 µM and MBC values of 50 µM, and had no antimicrobial activity toward all tested gram-negative strains (Table S2).

**TABLE 1:** Relative antimicrobial activities of mycoidesin and nisin A against *Listeria monocytogenes* strains

Strain^*a*^	Serotype	MIC (µM)	
		Mycoidesin	Nisin A
*L. monocytogenes* ATCC 19111	1/2a	0.39	3.13
*L. monocytogenes* ATCC 15313	1/2a	0.78	3.13
*L. monocytogenes* CMCC54002	1/2a	0.78	6.25
*L. monocytogenes* CICC 21632	1/2b	0.20	1.56
*L. monocytogenes* ATCC 51780	1/2b	0.39	3.13
*L. monocytogenes* ATCC BAA-751	1/2b	0.39	3.13
*L. monocytogenes* ATCC 19112	1/2c	0.39	1.56
*L. monocytogenes* ATCC 7644	1/2c	0.78	3.13
*L. monocytogenes* ATCC 19113	3a	0.39	3.13
*L. monocytogenes* ATCC 19114	4a	0.39	1.56
*L. monocytogenes* LM201	4b	0.39	3.13
*L. monocytogenes* LM605	4b	0.39	6.25
*L. monocytogenes* ATCC 19115	4b	0.78	6.25
*L. monocytogenes* ATCC 13932	4b	0.39	3.13
*L. monocytogenes* ATCC 19116	4c	0.78	3.13
*L. monocytogenes* ATCC 19117	4d	0.78	3.13
*L. monocytogenes* ATCC 19118	4e	0.78	3.13
*L. monocytogenes* NCT0890	7	0.78	3.13

^*a*^ *L. monocytogenes* LM201 and LM605 were kindly provided by Dr. Mei Liu (Huazhong Agricultural University, Wuhan, China). ATCC, American Type Culture Collection. CMCC, China Medical Culture Collection. CICC, China Center of Industrial Culture Collection. NCTC, National Collection of Type Cultures.

### Mode of action of mycoidesin

For bactericidal substances, the MBC is usually one to four times greater than the MIC, whereas for bacteriostatic substances, the MBC is many times greater than the MIC (30). Based on the MIC and MBC results, we hypothesized that mycoidesin exhibits a bacteriostatic effect against *L. monocytogenes* and a bactericidal effect against other indicator bacteria. Therefore, we used *L. monocytogenes* ATCC19111 and *B. cereus* CMCC63301 to evaluate the antimicrobial mechanism of mycoidesin.

**Fig 4:**
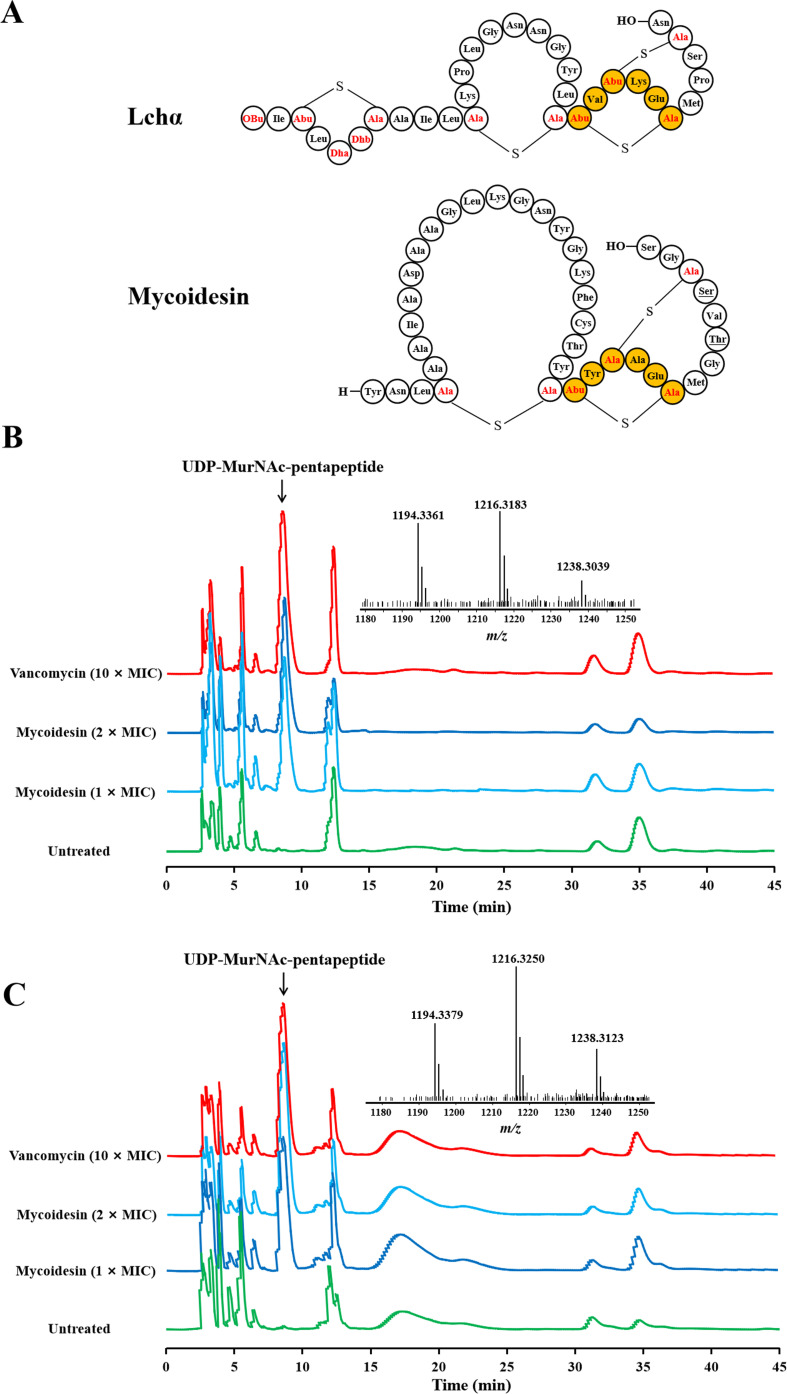
Mycoidesin blocks cell wall synthesis in *Listeria monocytogenes* ATCC19111 and *Bacillus cereus* CMCC63301. (A) Primary structure of Lchα and mycoidesin. Lipid II-binding domains are marked in orange. (B) Intracellular accumulation of the cell wall precursor UDP-MurNAc pentapeptide in mycoidesin-treated *L. monocytogenes* ATCC19111. (C) Intracellular accumulation of the cell wall precursor UDP-MurNAc pentapeptide in mycoidesin-treated cells of *B. cereus* CMCC63301. Vancomycin (10× MIC), a known inhibitor of peptidoglycan synthesis, was used as a positive control. The UDP-MurNAc pentapeptide was identified via mass spectrometry analysis. The calculated molecular mass (monoisotopic) was 1,193.34 Da, and singly charged ions and multiform sodium salts were detected.

Structural analysis showed that mycoidesin contained the same putative Lipid II-binding site as Lchα (Fig. 4A) (31). Mycoidesin may block the synthesis of sensitive bacterial cell walls by binding to peptidoglycan precursor Lipid II. This inference can be verified by detecting the intracellular accumulation of peptidoglycan precursor, UDP-MurNAc-pentapeptide, following treatment with antimicrobial substances (27, 32). Intracellular accumulation of UDP-MurNAc-pentapeptide was observed in both *L. monocytogenes* and *B. cereus* cells treated with vancomycin (a known peptidoglycan synthesis inhibitor) and mycoidesin (Fig. 4B and C). This result confirms that mycoidesin can block the synthesis of cell walls in sensitive bacteria.

**Fig 5:**
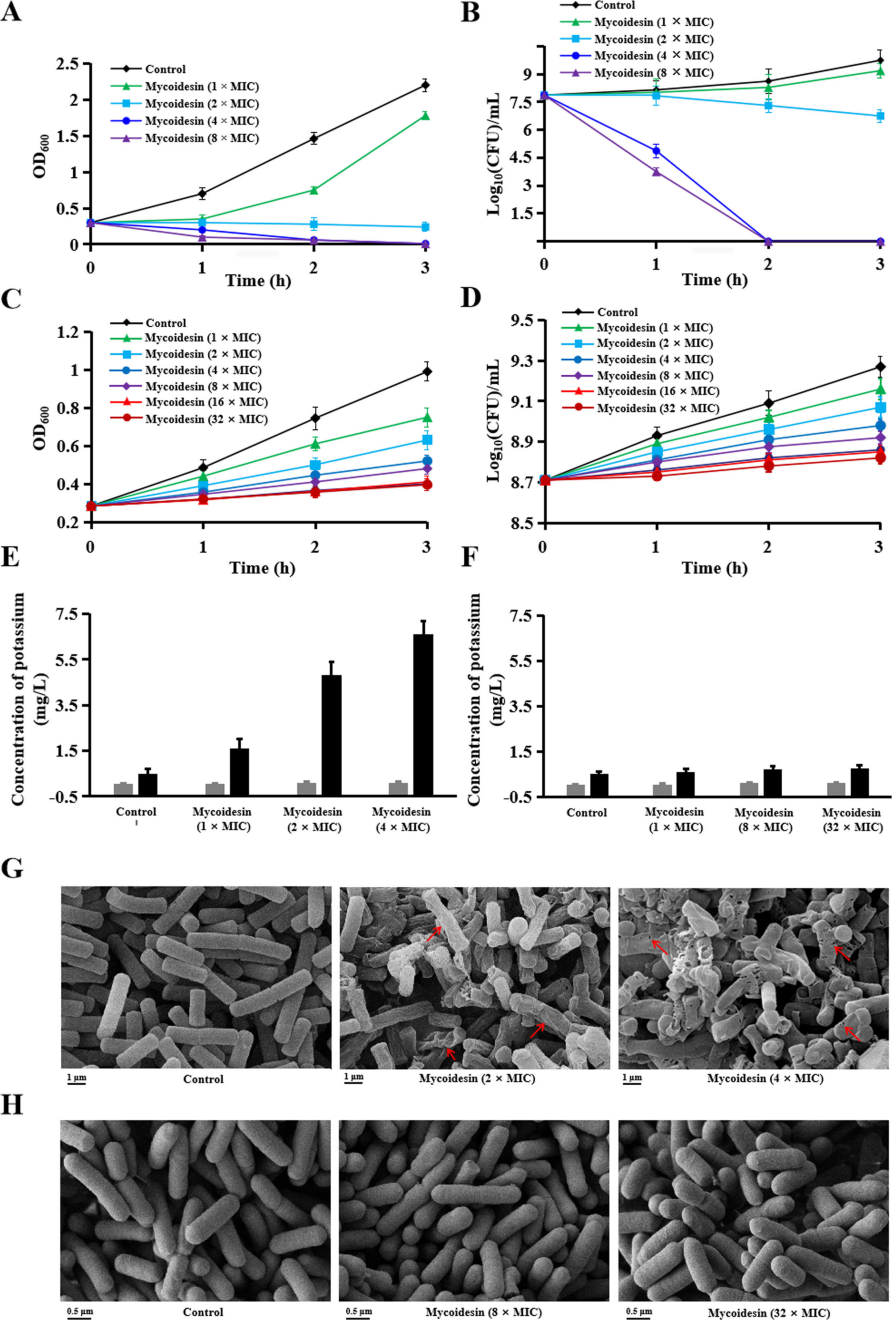
Mode of action of mycoidesin on *Listeria monocytogenes* and *Bacillus cereus*. (A) Effect of mycoidesin on the optical density of *B. cereus* CMCC63301 cultures. (B) Effect of mycoidesin on the viable cell count of *B. cereus* CMCC63301. (C) Effect of mycoidesin on the optical density of *L. monocytogenes* ATCC19111 culture. (D) Effect of mycoidesin on the viable count of *L. monocytogenes* ATCC19111. (E) Potassium release from *B. cereus* CMCC63301 cells treated with 1×, 2×, and 4× the MIC of mycoidesin. (F) Potassium release from *L. monocytogenes* ATCC19111 cells treated with mycoidesin at 1×, 8×, and 32× MIC. (G) SEM observation of *B. cereus* CMCC63301 cells treated with 2× and 4× MIC of mycoidesin. (H) SEM observation of *L. monocytogenes* ATCC19111 cells treated with 8× and 32× MIC of mycoidesin.

In addition, cell membrane damage and death of sensitive bacteria were detected following treatment with mycoidesin. At a concentration of 1× MIC, mycoidesin suppressed the growth of *B. cereus* ATCC14579; at 2× MIC, mycoidesin completely inhibited its growth; at 4× and 8× MIC, mycoidesin caused cell death after 2 h of treatment (Fig. 5A and B). Moreover, the potassium release assay and scanning electron microscopy (SEM) observations indicated that mycoidesin caused damage to the cell membrane of *B. cereus* (Fig. 5E). In contrast, the growth of *L. monocytogenes* ATCC19111 was suppressed by mycoidesin in a time- and concentration-dependent manner (Fig. 5C); however, mycoidesin did not cause *L. monocytogenes* cell membrane damage or cell death, even at high concentrations (32× MIC) (Fig. 5D, F, and H).

Collectively, these results indicate that mycoidesin exhibits a bacteriostatic effect rather than a bactericidal effect on *L. monocytogenes* both at low and high concentrations. This bacteriostatic effect is mediated by blocking cell wall synthesis through binding to Lipid II, thus inhibiting the growth of *L. monocytogenes*. In other sensitive strains, such as *B. cereus*, mycoidesin exerts a bacteriostatic effect at low concentrations through the same mechanism. In contrast, at high concentrations, it exerts a bactericidal effect, which can damage the cell membrane and ultimately cause cell death.

### Stability of mycoidesin

**Fig 6:**
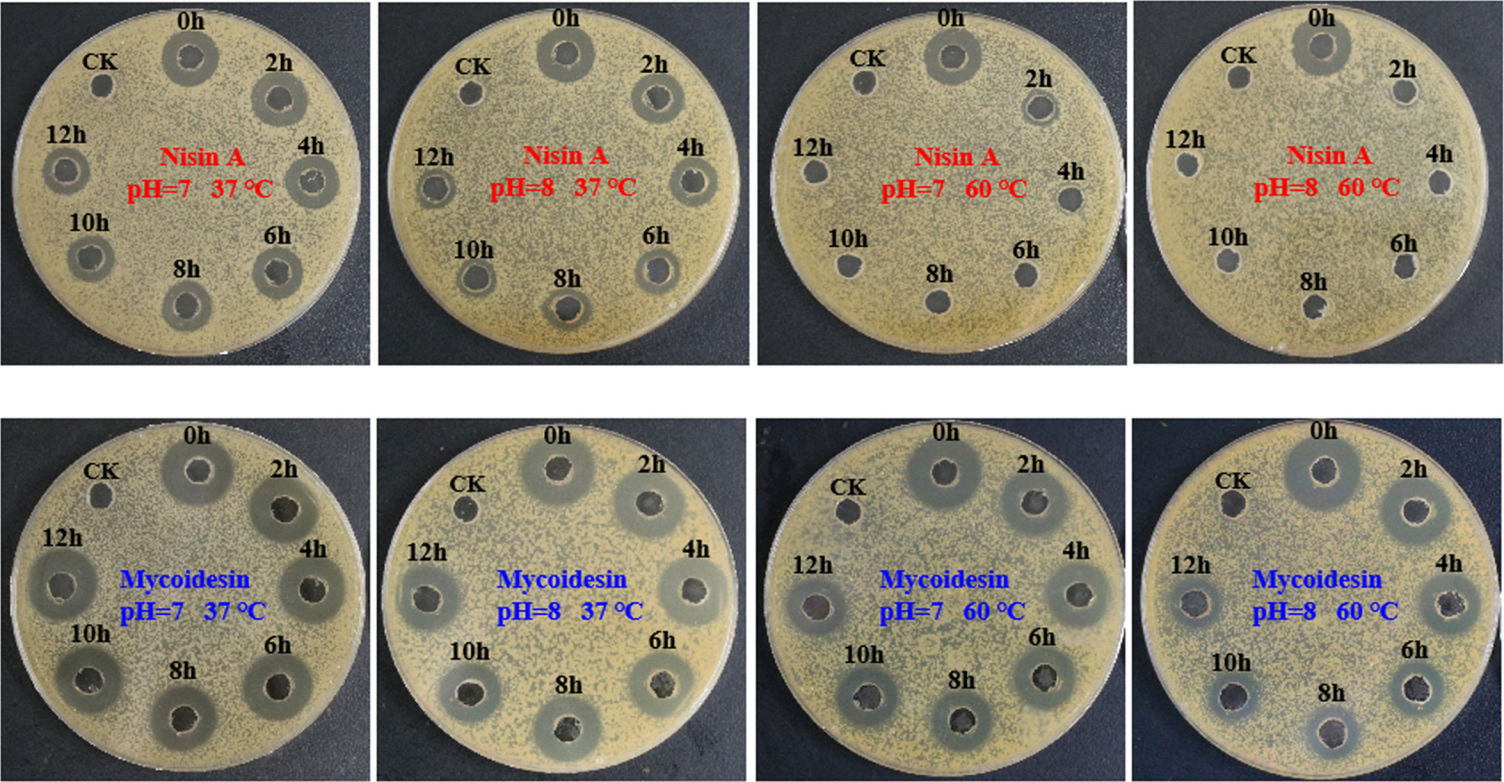
Relative stabilities of mycoidesin and nisin A. The agar plate diffusion method was used to detect the residual activity of the antimicrobial substances after treatment. *Bacillus subtilis* CMCC63501 was used as an indicator.

The relative stabilities of mycoidesin and nisin A were tested using the diffusion method. Under pH 7.0 and 37°C conditions, the antimicrobial activities of mycoidesin remained unchanged, whereas those of nisin A decreased with increasing incubation time (Fig. 6). Under pH 8.0 and 37°C conditions, the antimicrobial activities of both mycoidesin and nisin A decreased; however, the reduction in nisin A activity was significantly greater than that of mycoidesin. Under pH 7.0 and 60°C, as well as pH 8.0 and 60°C conditions, the antimicrobial activity of mycoidesin decreased with increasing incubation time. In contrast, nisin A lost most of its activity after 2 h of treatment and was completely inactivated after 4 h of exposure. These results indicate that mycoidesin is more stable compared to nisin A.

### Cytotoxic and hemolytic activities of mycoidesin

**Fig 7:**
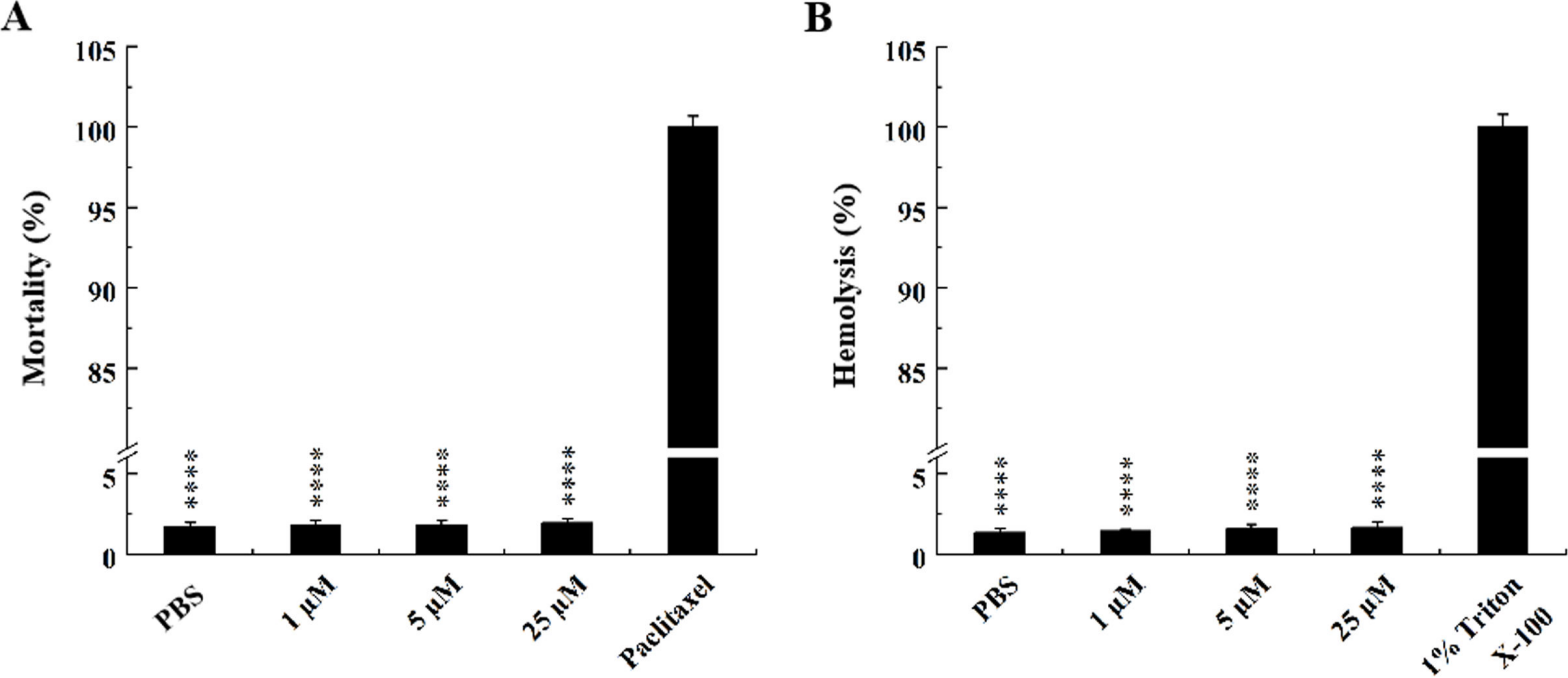
Cytotoxicity and hemolytic activities of mycoidesin. (A) Mycoidesin cytotoxicity in NIH-3T3 cells. PBS and paclitaxel were used as positive and negative controls, respectively. (B) Hemolytic activity of mycoidesin in defibrillated sheep blood cells. PBS and Triton X-100 (1%) were used as negative and positive controls, respectively. ****P ≤ 0.0001.

The cytotoxic and hemolytic activities of mycoidesin were measured in NIH-3T3 and defibrillated sheep blood cells, respectively. Mycoidesin showed low cytotoxic (1.85%) and hemolytic activities (0.9%) even at high concentrations (25 µM), indicating its biosafety (Fig. 7).

### Mycoidesin can be efficiently used for the control of *L. monocytogenes* in beef

**Fig 8:**
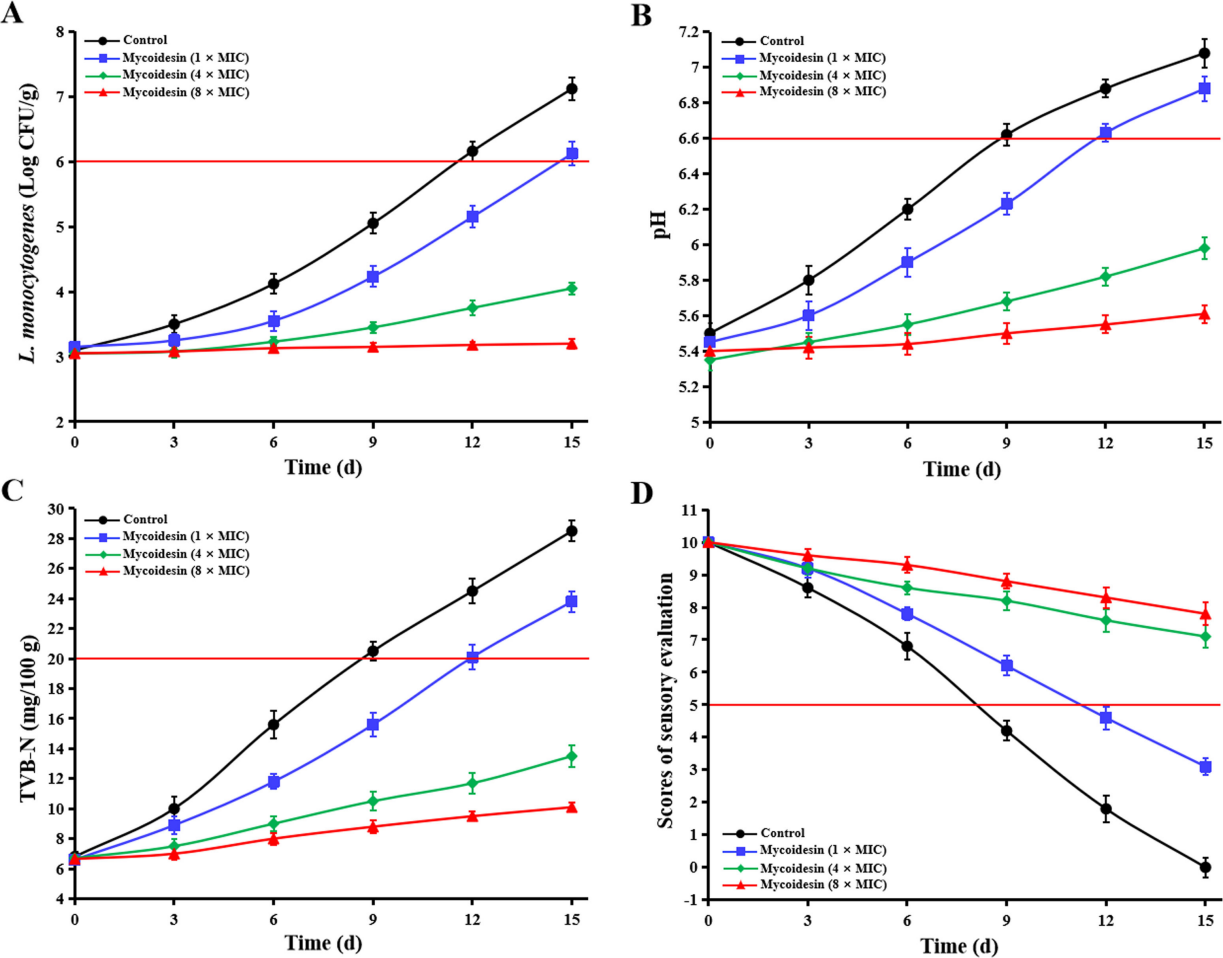
Effect of mycoidesin on *L. monocytogenes* counts (A), pH (B), TVB-N (C), and sensory evaluation (D) of beef during the 15-day storage period. The thresholds of 6× log CFU/g meat, pH 6.6, 20 mg/100 g meat, and a score of sensory evaluation 5.0 are indicated by red lines.

The ability of mycoidesin to preserve beef was evaluated using microbiological, pH, TVB-N, and sensory analyses. The total bacterial count of *L. monocytogenes*, as well as the values of pH and TVB-N, of all test groups increased as the storage period increased, and the values of the negative control group were consistently higher than those of the treatment group, while those of sensory evaluation decreased, with the negative control group’s values consistently lower than the treatment group’s (Fig. 8). The total bacterial count of *L. monocytogenes* at 12 days, pH value at 9 days, TVB-N value at 9 days, and sensory evaluation value at 9 days of the negative control group exceeded the thresholds (6 × log CFU/g meat, pH 6.6, 20 mg/100 g meat, and 5.0, respectively) (33). In contrast, the thresholds were exceeded in the 1× MIC mycoidesin group on days 15, 12, 12, and 12, respectively. The 4× MIC and 8× MIC mycoidesin groups did not exceed the threshold on day 15 (Fig. 8). These results indicate that mycoidesin effectively inhibited the growth of *L. monocytogenes* in beef and delayed the decline in beef quality.

## DISCUSSION

In this study, we used *L. monocytogenes* as the indicator bacterium to screen *B. cereus* group strains from soil samples collected from various regions and habitats across China. We screened the *B. mycoides* LX30 strain and identified mycoidesin as its antimicrobial component. Aside from the LX30 strain, 12 of the 132 strains screened in this study also showed antimicrobial activities against *L. monocytogenes*. Genomic sequencing and bacteriocin synthesis gene clusters revealed that anti-*L*. *monocytogenes* substances may be novel lantibiotics, cyclic peptides, and sactibiotics, which will be investigated in future studies.

The bacteriocin-producing bacteria used in this study, *B. mycoides*, are psychrotolerant members of the *B. cereus* group. Most related research has mainly focused on promoting plant growth and enhancing plant stress tolerance; however, reports on bacteriocins are scarce (34–37). Only Sharma and Gautam isolated a *B. mycoides* strain from whey; it produced bacteriocin with strong antimicrobial activities against *L. monocytogenes* and *Leuconostoc mesenteroides*; however, the molecular weight, structure, and synthetic gene cluster of the bacteriocin were not identified (38). To the best of our knowledge, mycoidesin, whose structure and synthetic gene cluster were determined in the present study, is the first identified bacteriocin of *B. mycoides*.

The number of *L. monocytogenes* strains used as indicator bacteria reached 18, and their serotypes covered 10 out of 13 serotypes of *L. monocytogenes.* Multiple strains belonged to serotypes 4b, 1/2b, and 1/2a, which are responsible for most human listeriosis cases (1). The MIC test results showed that mycoidesin exhibited 4- to 16-fold higher bacteriostatic activity against these tested *L. monocytogenes* strains than nisin A, highlighting its potential application in combating *L. monocytogenes*. In addition, mycoidesin exerted antimicrobial activity against other food-borne pathogens, such as *B. cereus*, *S. aureus*, and *C. perfringens*, among others. Our analysis of the mode of action of mycoidesin revealed that it blocked the synthesis of *L. monocytogenes* and *B. cereus* cell walls by binding to Lipid II, thereby inhibiting their growth. Mycoidesin did not cause cell membrane damage or cell death in *L. monocytogenes*; however, it caused cell membrane damage and cell death in *B. cereus*. The MIC and MBC results indicated that mycoidesin exhibits a bactericidal mechanism similar to that of *B. cereus* against other sensitive bacteria, such as *C. perfringens* and *S. aureus*. We speculated that the difference in these antimicrobial mechanisms is due to variations in the membrane thickness of different strains. Mycoidesin is a shorter peptide with three internal lanthionine rings, and the cell membrane of *L. monocytogenes* is thicker than that of *B. cereus* and *S. aureus* (39–41). Thus, mycoidesin is not sufficiently long to span the cytoplasmic membrane of *L. monocytogenes*; hence, it does not damage the cell membrane of *L. monocytogenes* or cause cell death. Previous studies have reported a similar scenario on the lantibiotic gallidermin, which is a shorter peptide with four internal rings that formed pores in *Micrococcal* and *Staphylococcal* strains; however, it did not form pores in the *Lactococcus lactis* strain (42). Artificial liposome membrane assays confirmed that its pore-forming capacity was dependent on the membrane thickness. The lantibiotic NVB302, a shorter peptide, did not span the membrane and could not form membrane water channels (43). The relationship between the pore-forming capacity of mycoidesin and the cell membrane thickness of sensitive strains or other factors will be validated in future studies.

The application of nisin A as the natural food preservative of bacteriocins is greatly limited by its poor stability in neutral and alkaline conditions (15, 16). In this study, we tested and compared the stabilities of mycoidesin and nisin A under neutral, alkaline, and high-temperature conditions. The results showed that the stability of mycoidesin was significantly higher than that of nisin A under the same conditions, and the good stability of mycoidesin demonstrates its potential for practical applications.

The biosafety of bacteriocins is essential for their use as food preservatives. Currently, some bacteriocins produced by pathogens, such as streptolysin S, cytolysin, and BacSp222 from Group A *streptococcus*, *Enterococcus faecalis,* and *Staphylococcus pseudintermedius,* respectively, possess virulence factors (44–46). In this study, we determined the cytotoxic effects of mycoidesin on NIH-3T3 cells and the hemolytic capability of mycoidesin on defibrillated sheep blood. Mycoidesin exhibited low cytotoxic and hemolytic activities at low and high concentrations, preliminarily proving its biological safety. More biological safety tests, such as acute and chronic toxicity tests, as well as teratogenic, carcinogenic, mutagenic, and genotoxicity tests, will be performed in subsequent experiments.

Listeriosis outbreaks are associated with the consumption of food contaminated with *L. monocytogenes*. Meat and meat products have been reported to be the main food vectors for the transmission of *L. monocytogenes* to humans (5). In this study, we tested the ability of mycoidesin to preserve beef using microbiological, pH, TVB-N, and sensory analyses. The measurement results indicated that mycoidesin effectively inhibited the growth of *L. monocytogenes* in beef samples and delayed the decline in beef quality (Fig. 8). However, beef contains native microorganisms, and the species and quantities of these microorganisms vary significantly across beef from different sources. The presence or absence of native microorganisms significantly impacts the final effectiveness of preservatives. In this study, the application of mycoidesin in beef preservation was conducted under the condition of removing native microorganisms and inoculating a fixed amount of *L. monocytogenes*. Although there are certain differences between the experimental design in this study and the practical application of preservatives in beef preservation, our findings demonstrated the effectiveness of mycoidesin in controlling *L. monocytogenes* in beef. In subsequent experiments, we will also consider the influence of native microorganisms to obtain results that more accurately reflect practical application conditions.

## Data Availability

The genome sequence of *B. mycoides* LX30 and the sequence of the mycoidesin gene cluster were deposited in GenBank with accession numbers GCA_022532085.1 and PQ272043, respectively.

## SUPPLEMENTAL MATERIAL

Supplemental tables aem.00067-25-s0001.doc Tables S1 and S2.

ASM does not own the copyrights to Supplemental Material that may be linked to, or accessed through, an article. The authors have granted ASM a non-exclusive, world-wide license to publish the Supplemental Material files. Please contact the corresponding author directly for reuse.

## References

[1] Bhunia AK. 2018. Foodborne microbial pathogens: mechanisms and pathogenesis. 2nd ed. Springer, New York.

[2] Liu X, Xia X, Liu Y, Li Z, Shi T, Zhang H, Dong Q. 2024. Recent advances on the formation, detection, resistance mechanism, and control technology of Listeria monocytogenes biofilm in food industry. Food Res Int 180:114067. doi:10.1016/j.foodres.2024.114067 38395584

[3] Bodie AR, O’Bryan CA, Olson EG, Ricke SC. 2023. Natural antimicrobials for Listeria monocytogenes in ready-to-eat meats: current challenges and future prospects. Microorganisms 11:1301. doi:10.3390/microorganisms11051301 37317275 PMC10222861

[4] Datta AR, Burall LS. 2018. Serotype to genotype: the changing landscape of listeriosis outbreak investigations. Food Microbiol 75:18–27. doi:10.1016/j.fm.2017.06.013 30056958

[5] Grigore-Gurgu L, Bucur FI, Mihalache OA, Nicolau AI. 2024. Comprehensive review on the biocontrol of Listeria monocytogenes in food products. Foods 13:734. doi:10.3390/foods13050734 38472848 PMC10931214

[6] Ngema SS, Madoroba E. 2024. A mini-review of anti-Listerial compounds from marine actinobacteria (1990–2023). Antibiotics (Basel) 13:362. doi:10.3390/antibiotics13040362 38667038 PMC11047329

[7] Khorshidian N, Khanniri E, Mohammadi M, Mortazavian AM, Yousefi M. 2021. Antibacterial activity of pediocin and pediocin-producing bacteria against Listeria monocytogenes in meat products. Front Microbiol 12:709959. doi:10.3389/fmicb.2021.709959 34603234 PMC8486284

[8] Li W, Bai L, Fu P, Han H, Liu J, Guo Y. 2018. The epidemiology of Listeria monocytogenes in China. Foodborne Pathog Dis 15:459–466. doi:10.1089/fpd.2017.2409 30124341

[9] Koopmans MM, Brouwer MC, Vázquez-Boland JA, van de Beek D. 2023. Human listeriosis. Clin Microbiol Rev 36:e0006019. doi:10.1128/cmr.00060-19 36475874 PMC10035648

[10] Bruna GOL, Thais ACC, Lígia ACC. 2018. Food additives and their health effects: a review on preservative sodium benzoate. Afr J Biotechnol 17:306–310. doi:10.5897/AJB2017.16321

[11] Silva MM, Lidon FC. 2016. Food preservatives - an overview on applications and side effects. Emir J Food Agric 28:366. doi:10.9755/ejfa.2016-04-351

[12] Johnson EM, Jung D-G, Jin D-Y, Jayabalan DR, Yang DSH, Suh JW. 2018. Bacteriocins as food preservatives: challenges and emerging horizons. Crit Rev Food Sci Nutr 58:2743–2767. doi:10.1080/10408398.2017.1340870 28880573

[13] O’Connor PM, Ross RP, Hill C, Cotter PD. 2015. Antimicrobial antagonists against food pathogens: a bacteriocin perspective. Curr Opin Food Sci 2:51–57. doi:10.1016/j.cofs.2015.01.004

[14] Sugrue I, Ross RP, Hill C. 2024. Bacteriocin diversity, function, discovery and application as antimicrobials. Nat Rev Microbiol 22:556–571. doi:10.1038/s41579-024-01045-x 38730101 PMC7616364

[15] Garg N, Tang W, Goto Y, Nair SK, van der Donk WA. 2012. Lantibiotics from Geobacillus thermodenitrificans. Proc Natl Acad Sci USA 109:5241–5246. doi:10.1073/pnas.1116815109 22431611 PMC3325677

[16] Rollema HS, Kuipers OP, Both P, de Vos WM, Siezen RJ. 1995. Improvement of solubility and stability of the antimicrobial peptide nisin by protein engineering. Appl Environ Microbiol 61:2873–2878. doi:10.1128/aem.61.8.2873-2878.1995 7487019 PMC167563

[17] Morandini L, Caulier S, Bragard C, Mahillon J. 2024. Bacillus cereus sensu lato antimicrobial arsenal: an overview. Microbiol Res 283:127697. doi:10.1016/j.micres.2024.127697 38522411

[18] Deng S, Liu S, Li X, Liu H, Li F, Liu K, Zeng H, Zeng X, Xin B. 2022. Thuricins: novel leaderless bacteriocins with potent antimicrobial activity against Gram-positive foodborne pathogens. J Agric Food Chem 70:9990–9999. doi:10.1021/acs.jafc.2c02890 35924350

[19] Xin B, Zheng J, Xu Z, Song X, Ruan L, Peng D, Sun M. 2015. The Bacillus cereus group is an excellent reservoir of novel lanthipeptides. Appl Environ Microbiol 81:1765–1774. doi:10.1128/AEM.03758-14 25548056 PMC4325169

[20] Xin B, Liu H, Zheng J, Xie C, Gao Y, Dai D, Peng D, Ruan L, Chen H, Sun M. 2020. In silico analysis highlights the diversity and novelty of circular bacteriocins in sequenced microbial genomes. mSystems 5:e00047-20. doi:10.1128/mSystems.00047-20 32487738 PMC8534725

[21] He Z, Kisla D, Zhang L, Yuan C, Green-Church KB, Yousef AE. 2007. Isolation and identification of a Paenibacillus polymyxa strain that coproduces a novel lantibiotic and polymyxin. Appl Environ Microbiol 73:168–178. doi:10.1128/AEM.02023-06 17071789 PMC1797129

[22] Liu H, Xin B, Zheng J, Zhong H, Yu Y, Peng D, Sun M. 2022. Build a bioinformatic analysis platform and apply it to routine analysis of microbial genomics and comparative genomics. Protoc Exch. doi:10.21203/rs.2.21224/v6

[23] Blin K, Shaw S, Augustijn HE, Reitz ZL, Biermann F, Alanjary M, Fetter A, Terlouw BR, Metcalf WW, Helfrich EJN, van Wezel GP, Medema MH, Weber T. 2023. antiSMASH 7.0: new and improved predictions for detection, regulation, chemical structures and visualisation. Nucleic Acids Res 51:W46–W50. doi:10.1093/nar/gkad344 37140036 PMC10320115

[24] van Heel AJ, de Jong A, Song C, Viel JH, Kok J, Kuipers OP. 2018. BAGEL4: a user-friendly web server to thoroughly mine RiPPs and bacteriocins. Nucleic Acids Res 46:W278–W281. doi:10.1093/nar/gky383 29788290 PMC6030817

[25] Wu YB, Zhou LB, Lu FX, Bie XM, Zhao HZ, Zhang C, Lu ZX, Lu YJ. 2019. Discovery of a novel antimicrobial lipopeptide, brevibacillin V, from Brevibacillus laterosporus fmb70 and its application on the preservation of skim milk. J Agric Food Chem 67:12452–12460. doi:10.1021/acs.jafc.9b04113 31674183

[26] Xin B, Zheng J, Xu Z, Li C, Ruan L, Peng D, Sun M. 2015. Three novel lantibiotics, ticins A1, A3, and A4, have extremely stable properties and are promising food biopreservatives. Appl Environ Microbiol 81:6964–6972. doi:10.1128/AEM.01851-15 26231642 PMC4579438

[27] Ling LL, Schneider T, Peoples AJ, Spoering AL, Engels I, Conlon BP, Mueller A, Schäberle TF, Hughes DE, Epstein S, Jones M, Lazarides L, Steadman VA, Cohen DR, Felix CR, Fetterman KA, Millett WP, Nitti AG, Zullo AM, Chen C, Lewis K. 2015. A new antibiotic kills pathogens without detectable resistance. Nature 517:455–459. doi:10.1038/nature14098 25561178 PMC7414797

[28] Baindara P, Chaudhry V, Mittal G, Liao LM, Matos CO, Khatri N, Franco OL, Patil PB, Korpole S. 2016. Characterization of the antimicrobial peptide penisin, a class Ia novel lantibiotic from Paenibacillus sp. strain A3. Antimicrob Agents Chemother 60:580–591. doi:10.1128/AAC.01813-15 26574006 PMC4704198

[29] Hanchi H, Hammami R, Fernandez B, Kourda R, Ben Hamida J, Fliss I. 2016. Simultaneous production of formylated and nonformylated Enterocins L50A and L50B as well as 61A, a new glycosylated durancin, by Enterococcus durans 61A, a strain isolated from artisanal fermented milk in Tunisia. J Agric Food Chem 64:3584–3590. doi:10.1021/acs.jafc.6b00700 27111259

[30] Levison ME, Levison JH. 2009. Pharmacokinetics and pharmacodynamics of antibacterial agents. Infect Dis Clin North Am 23:791–815. doi:10.1016/j.idc.2009.06.008 19909885 PMC3675903

[31] Shenkarev ZO, Finkina EI, Nurmukhamedova EK, Balandin SV, Mineev KS, Nadezhdin KD, Yakimenko ZA, Tagaev AA, Temirov YV, Arseniev AS, Ovchinnikova TV. 2010. Isolation, structure elucidation, and synergistic antibacterial activity of a novel two-component lantibiotic lichenicidin from Bacillus licheniformis VK21. Biochemistry 49:6462–6472. doi:10.1021/bi100871b 20578714

[32] Schneider T, Kruse T, Wimmer R, Wiedemann I, Sass V, Pag U, Jansen A, Nielsen AK, Mygind PH, Raventós DS, Neve S, Ravn B, Bonvin A, De Maria L, Andersen AS, Gammelgaard LK, Sahl H-G, Kristensen H-H. 2010. Plectasin, a fungal defensin, targets the bacterial cell wall precursor Lipid II. Science 328:1168–1172. doi:10.1126/science.1185723 20508130

[33] Zhao D, Wang Q, Lu F, Bie X, Zhao H, Lu Z, Lu Y. 2022. A novel class IIb bacteriocin-plantaricin EmF effectively inhibits Listeria monocytogenes and extends the shelf life of beef in combination with chitosan. J Agric Food Chem 70:2187–2196. doi:10.1021/acs.jafc.1c06269 35019260

[34] Ali B, Wang X, Saleem MH, Azeem MA, Afridi MS, Nadeem M, Ghazal M, Batool T, Qayyum A, Alatawi A, Ali S. 2022. Bacillus mycoides PM35 reinforces photosynthetic efficiency, antioxidant defense, expression of stress-responsive genes, and ameliorates the effects of salinity stress in maize. Life (Basel) 12:219. doi:10.3390/life12020219 35207506 PMC8875943

[35] Fiedoruk K, Drewnowska JM, Mahillon J, Zambrzycka M, Swiecicka I. 2021. Pan-genome portrait of Bacillus mycoides provides insights into the species ecology and evolution. Microbiol Spectr 9:e0031121. doi:10.1128/spectrum.00311-21 34287030 PMC8552610

[36] Kurniawan A, Chuang HW. 2022. Rhizobacterial Bacillus mycoides functions in stimulating the antioxidant defence system and multiple phytohormone signalling pathways to regulate plant growth and stress tolerance. J Appl Microbiol 132:1260–1274. doi:10.1111/jam.15252 34365711

[37] Shahzad A, Aslam U, Ferdous S, Qin M, Siddique A, Billah M, Naeem M, Mahmood Z, Kayani S. 2024. Combined effect of endophytic Bacillus mycoides and rock phosphate on the amelioration of heavy metal stress in wheat plants. BMC Plant Biol 24:125. doi:10.1186/s12870-024-04812-3 38373884 PMC10877812

[38] Sharma N, Gautam N. 2008. Antibacterial activity and characterization of bacteriocin of Bacillus mycoides isolated from whey. Ind J Biotechnol 8:117–121.

[39] Ghosh BK, Carroll KK. 1968. Isolation, composition, and structure of membrane of Listeria monocytogenes. J Bacteriol 95:688–699. doi:10.1128/jb.95.2.688-699.1968 4966553 PMC252066

[40] Nickels JD, Chatterjee S, Stanley CB, Qian S, Cheng X, Myles DAA, Standaert RF, Elkins JG, Katsaras J. 2017. The in vivo structure of biological membranes and evidence for lipid domains. PLoS Biol 15:e2002214. doi:10.1371/journal.pbio.2002214 28542493 PMC5441578

[41] Joodaki F, Martin LM, Greenfield ML. 2022. Generation and computational characterization of a complex Staphylococcus aureus lipid bilayer. Langmuir 38:9481–9499. doi:10.1021/acs.langmuir.2c00483 35901279

[42] Bonelli RR, Schneider T, Sahl HG, Wiedemann I. 2006. Insights into in vivo activities of lantibiotics from gallidermin and epidermin mode-of-action studies. Antimicrob Agents Chemother 50:1449–1457. doi:10.1128/AAC.50.4.1449-1457.2006 16569864 PMC1426925

[43] Pokhrel R, Bhattarai N, Baral P, Gerstman BS, Park JH, Handfield M, Chapagain PP. 2022. Lipid II binding and transmembrane properties of various antimicrobial lanthipeptides. J Chem Theory Comput 18:516–525. doi:10.1021/acs.jctc.1c00666 34874159

[44] Datta V, Myskowski SM, Kwinn LA, Chiem DN, Varki N, Kansal RG, Kotb M, Nizet V. 2005. Mutational analysis of the group A streptococcal operon encoding streptolysin S and its virulence role in invasive infection. Mol Microbiol 56:681–695. doi:10.1111/j.1365-2958.2005.04583.x 15819624

[45] Van Tyne D, Martin MJ, Gilmore MS. 2013. Structure, function, and biology of the Enterococcus faecalis cytolysin. Toxins (Basel) 5:895–911. doi:10.3390/toxins5050895 23628786 PMC3709268

[46] Wladyka B, Piejko M, Bzowska M, Pieta P, Krzysik M, Mazurek Ł, Guevara-Lora I, Bukowski M, Sabat AJ, Friedrich AW, Bonar E, Międzobrodzki J, Dubin A, Mak P. 2015. A peptide factor secreted by Staphylococcus pseudintermedius exhibits properties of both bacteriocins and virulence factors. Sci Rep 5:14569. doi:10.1038/srep14569 26411997 PMC4585962

