# Supplementary material for: Mycoidesin, a novel lantibiotic, exhibits potent bacteriostatic activity against Listeria monocytogenes and effectively controls its growth in beef
Source: Appl Environ Microbiol. 2025 Mar 25;91(4):e00067-25. doi: 10.1128/aem.00067-25 (PMC12016531; doi:10.1128/aem.00067-25)
Supplement: Supplemental tables — Tables S1 and S2. [file aem.00067-25-s0001.doc]

**Table S1 A summary of the fragment ions in the MS/MS spectrum of mycoidesin**

| Fragment ion | Measured (*m/z*) | Calculated (*m/z*) |
| --- | --- | --- |
| y1 | 106.0508 | 106.0504 |
| a1 | 136.071 | 136.0655 |
| y2 | 163.0696 | 163.0718 |
| b2 | 278.1108 | 278.1141 |
| b3 | 391.1946 | 391.1981 |
| b21 | 1056.0256^2+^ | 1056.0231^2+^ |
| b33 | 1645.2178^2+^ | 1645.2328^2+^ |
| b34 | 1116.1650^3+^ | 1116.1518^3+^ |
| y32 | 1531.1697^2+^ | 1531.1555^2+^ |
| y33 | 1587.7117^2+^ | 1587.6842^2+^ |
| y34 | 1096.8161^3+^ | 1096.8247^3+^ |

**Table S2 Antimicrobial activities of mycoidesin**

| **Strain^a^** | **Medium^b^** | **MIC (µM)^c^** | **MBC (µM)^c^** |
| --- | --- | --- | --- |
| **Gram-positive bacteria** |  |  |  |
| *Bacillus cereus* ATCC 14579 | LB | 3.13 | 3.13 |
| *Bacillus cereus* CMCC 63301 | LB | 1.56 | 3.13 |
| *Bacillus cereus* CMCC 63303 | LB | 1.56 | 1.56 |
| *L. monocytogenes* ATCC 19111 | TSB-YE | 0.39 | NA |
| *L.monocytogenes* ATCC 15313 | TSB-YE | 0.78 | NA |
| *L. monocytogenes* CMCC 54002 | TSB-YE | 0.78 | NA |
| *L. monocytogenes* CICC 21632 | TSB-YE | 0.20 | NA |
| *L. monocytogenes* ATCC 51780 | TSB-YE | 0.39 | NA |
| *L. monocytogenes* ATCC BAA-751 | TSB-YE | 0.39 | NA |
| *L. monocytogenes* ATCC 19112 | TSB-YE | 0.39 | NA |
| *L. monocytogenes* ATCC 7644 | TSB-YE | 0.78 | NA |
| *L. monocytogenes* ATCC 19113 | TSB-YE | 0.39 | NA |
| *L. monocytogenes* ATCC 19114 | TSB-YE | 0.39 | NA |
| *L. monocytogenes* LM201 | TSB-YE | 0.39 | NA |
| *L. monocytogenes* LM605 | TSB-YE | 0.39 | NA |
| *L. monocytogenes* ATCC 19115 | TSB-YE | 0.78 | NA |
| *L. monocytogenes* ATCC 13932 | TSB-YE | 0.39 | NA |
| *L. monocytogenes* ATCC 19116 | TSB-YE | 0.78 | NA |
| *L. monocytogenes* ATCC 19117 | TSB-YE | 0.78 | NA |
| *L. monocytogenes* ATCC 19118 | TSB-YE | 0.78 | NA |
| *L. monocytogenes* NCT0890 | TSB-YE | 0.78 | NA |
| *Clostridium perfringens* ATCC 13124 | RCM | 1.56 | 3.13 |
| *Bacillus subtilis* CMCC 63501 | LB | 1.56 | 3.13 |
| *Staphylococcus aureus* ATCC 43300 | NB | 25.00 | 50 |
| *Staphylococcus aureus* ATCC 6538 | NB | 25.00 | 50 |
| *Staphylococcus epidermdis* CMCC 26069 | NB | 25.00 | 50 |
| *Enterococcus faecalis* ATCC 29212 | BHI | 3.13 | 6.25 |
| *Streptococcus suis* SC19 | BHI | 6.25 | 12.5 |
| **Gram-negative bacteria** |  |  |  |
| *Pseudomonas aeruginosa* ATCC 27853 | NB | NA | NA |
| *Klebsiella pneumoniae* CMCC 46117 | NB | NA | NA |
| *Salmonella enterica* serotype Paratyphi CMCC 50093 | NB | NA | NA |
| *Shigella serogroups* CMCC 51105 | NB | NA | NA |
| *Escherichia coli* ATCC 25922 | NB | NA | NA |
| *Acinetobacter baumannii*ATCC 19606 | NB | NA | NA |
| *Salmonella Typhimurium* ATCC 14028 | NB | NA | NA |
| *Serratia marcescens* CMCC 41002 | NB | NA | NA |

*^a^*ATCC, American Type Culture Collection. CMCC, China Medical Culture Collection. CICC, [China Center of Industrial Culture Collection](http://english.china-cicc.org/). NCTC, National Collection of Type Cultures. *L. monocytogenes* LM201 and LM605 were kindly provided by Ph.D Mei Liu (Huazhong Agricultural University, Wuhan, China). *S. suis* SC19 was kindly provided by Ph.D Meilin Jin (Huazhong Agricultural University, Wuhan, China).

*^b^*LB, Luria-Bertani; TSB-YE, tryptic soy broth with yeast extract; RCM, reinforced *Clostridium* medium; NB, nutrient broth; BHI, brain heart infusion broth.

*^c^*NA, No activity was detected even at the highest concentration of mycoidesin (100 µM).
